# High-Grade Glioma Tumoral Cells in a Case of Postoperative, Recurrent Subdural Hematoma… Where Did They Come From?

**DOI:** 10.3390/curroncol33050283

**Published:** 2026-05-10

**Authors:** Paul E. Constanthin, Arthur Durouchoux, Gianpaolo Jannelli, Mégane Le Quang, Guillaume Chotard, Julien Engelhardt

**Affiliations:** 1 Department of Neurosurgery, Geneva University Hospitals (HUG), 1205 Geneva, Switzerland; 2 Faculty of Medicine, University of Geneva (UNIGE), 1205 Geneva, Switzerland; 3 Department of Neurosurgery, Pellegrin Hospital, Bordeaux University Hospital, 33000 Bordeaux, France; 4 Pathology Department, Bordeaux University Hospital, 33000 Bordeaux, France; 5 Institute of Cellular Biochemistry and Genetics, CNRS UMR 5095, Bordeaux University, 33076 Bordeaux, France

**Keywords:** high-grade glioma, subdural hematoma, tumoral cells dissemination, case report

## Abstract

*Background:* High-grade glioma (HGG), formerly known as Glioblastoma multiforme, can be complicated by hematomas, either at the initial presentation or after surgical removal. Rarely, postoperative bleeding can occur extra-axially, resulting in subdural hematomas (SDH) that might require surgical evacuation. Of note, little is known about the cellular composition of those hematomas. *Observations:* We present the case of a patient operated on for HGG who developed postoperative recurrent SDH that required multiple surgical evacuations. Histopathological analyses of the membranes comprising the SDH revealed the presence of HGG tumoral cells. *Conclusions:* Based on our observation, hematomas associated with HGG, either extra or intra-axial, should be suspected of being a sign of tumoral recurrence or spread and histopathological analyses might be considered as they could lead to further adaptation of the patient’s treatment.

## Simple Summary

High-grade gliomas can be complicated by hematomas requiring surgical evacuation. We present the case of a patient operated on for high-grade glioma who presented with postoperative recurrent subdural hematoma. Histopathological analyses revealed the presence of tumoral cells in the hematoma. Hematomas associated with a high-grade glioma could signal tumoral recurrence or spread, and histopathological analyses might be considered in these cases.

## 1. Introduction

High-grade glioma (HGG), formerly known as glioblastoma multiforme, is the most frequent primary malignant brain tumor and typically presents with symptoms such as neurological deficits, epileptic seizures or increased intracranial pressure when the lesion is sufficiently large or associated with significant perilesional edema [1,2,3]. More rarely, HGG might be discovered following the occurrence of concomitant, intracranial bleeding, resulting in intracranial hematomas (0.54–3.5%) [4]. Such hematomas appear to be mostly caused by tumoral bleeding of abnormal blood vessels [5]. However, extra-axial bleeding, whether associated with intracerebral hematoma or not, has also been reported in the literature [6,7,8].

Surgical removal of the lesion followed by adjuvant chemo- and radiotherapy (Stupp protocol) remains the gold-standard treatment of HGG [9]. Interestingly, postoperative bleeding of the surgical site resulting in hematoma can occur in approximately 2% of the cases [10], and rare cases of subdural hematomas (SDH) have been described in this context [11,12,13]. Such postoperative bleeding, sometimes associated with venous thromboembolism, are often associated with poorer prognosis [14]. Despite the description of such hemorrhagic complications, little is known about the cellular composition of those hematomas and whether they remain comparable to more classical SDH.

In this article, we present a case of recurrent, postoperative SDH in a patient initially operated on for HGG. Interestingly, histopathological analyses revealed the surprising presence of HGG-tumoral cells comparable to the original lesion in the membranes of the SDH obtained during surgical evacuation. This observation raises the question of the origin of the bleeding and whether the SDH had a role in the dissemination of tumoral cells outside the initial resection site.

## 2. Illustrative Case

**Figure 1:**
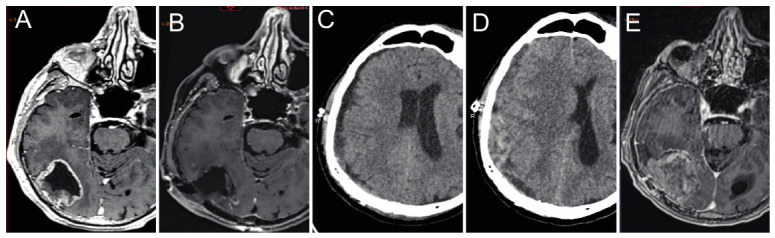
Representative images (MRI and CT scan) at different timepoints during patient’s management. (**A**) Preoperative T1 MRI with gadolinium injection showing a right occipital contrast-enhancing lesion with necrotic center and perilesional edema. (**B**) Postoperative T1 MRI with gadolinium injection confirming total resection of the contrast-enhancing component of the lesion without postoperative complication. (**C**) Postoperative CT scan after the evacuation of the first right-sided chronic SDH. (**D**) Emergency CT scan showing the recurrence of the right-sided SDH and its mass effect on the parenchyma (**E**) T1 MRI with gadolinium injection showing the recurrence of the right occipital lesion in the initial resection site.

**Figure 2:**
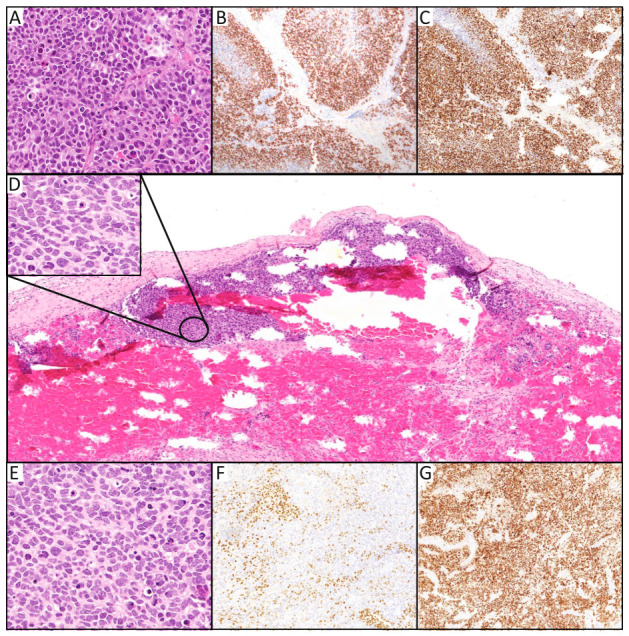
Immuno-histological analyses of the primary tumor and recurrent subdural hematoma. (**A**) The primary tumor consisted of a proliferation of undifferentiated cells with marked cytonuclear atypia (×40, H&E). (**B**,**C**) Immunohistochemistry of the primary tumor shows strong and diffuse expression of the glial marker OLIG2 (×10; (**B**)) and P53 (×10; (**C**)) in tumor cells. (**D**,**E**) Histological section, showing an organized hematoma with surrounding fibrous membrane. Clusters of atypical tumor cells are identified within the hematoma membrane and focally within the hematoma itself, supporting tumor involvement of the hematoma cavity. Analysis of the recurrent SDH and its membranes, obtained at a significant distance from the initial resection site, also displays a proliferation of undifferentiated cells with prominent cytonuclear atypia (×40, H&E). (**F**,**G**) Immunohistochemical staining of the recurrent SDH revealed focal expression of OLIG2 (×10; (**F**)) and diffuse expression of P53 (×10; (**G**)).

A 77 y.o. man with previously known high blood pressure, prostate cancer (considered in remission for years) and lumbar fixation presented with several months of visual impairment. A brain MRI showed a right, unifocal, contrast-enhancing (with necrotic center) occipital lesion measuring 4 × 4 cm with surrounding FLAIR hypersignal, suggesting a primary glial lesion (see Figure 1A). The patient was referred to our neurosurgical department, where a detailed neurological exam showed a left homonymous hemianopia with left-sided oculomotor apraxia. The mass was resected, and postoperative MRI confirmed total resection of the contrast-enhanced component of the lesion without complication (see Figure 1B). The patient received prophylactic anticoagulation during the rest of his hospital stay. Histopathological analyses revealed positivity for Cytokeratin E1/AE3, GFAP, Olig2, INSM1 and Synaptophysin. On the other hand, staining was negative for CK7, CK20, IDH-R132 and TTF1. The tumor showed strong overexpression of P53 and high Mib1 (90%) proliferation, while there was no loss of ATRX expression. Finally, molecular analysis revealed MGMT methylation, mutation of TERT promoter and the absence of genetic mutation or fusion in NGS testing. Therefore, the tumor was classified as WHO-Grade 4 HGG, MGMT-methylated, IDH Wild-type with Mib1 positivity in 90% of the cells (see Figure 2A–C). After multidisciplinary discussion in our dedicated neuro-oncological tumor board, adjuvant radio- and chemotherapy (Stupp protocol) adapted to the patient’s age was proposed and the patient was discharged without further complication on the fourth postoperative day (prophylactic anticoagulation was discontinued upon discharge).

Approximately 6 weeks after the surgery (before the start of the Stupp protocol), the patient suffered a traumatic fall at home and immediately developed a left hemiparesis followed by a decreased Glasgow coma scale (GCS) score from 15 to 12 (E3V4M5). An emergency CT scan revealed a voluminous right-sided (convexity) chronic SDH with a maximum size of 22 mm, responsible for a 12 mm shift of the midline. There was no abnormal coagulation status on laboratory analysis. An emergency surgery consisting of one burr hole centered on the SDH and the introduction of subdural drainage allowed for the evacuation of the hematoma. Partial re-expansion of the brain had already been observed during the surgery and was confirmed later on postoperative imaging (see Figure 1C). The subdural drainage was removed after 48 h without complication. No histopathological analysis was performed on the components of the SDH or on the patient’s cerebrospinal fluid (CSF). Postoperatively, the patient presented with a complete resolution of his condition and was discharged 7 days after the surgery (with GCS 15 and without any remaining motor disability). During the entire hospital stay, no anticoagulant treatment was administered to the patient.

The patient, however, presented a new neurological deterioration with left hemiparesis and decreased consciousness (GCS 10, E3V1M6) 2 days later, this time with no trauma history and without any abnormal coagulation status on laboratory analyses. Emergency CT scan and MRI were performed, revealing an acute recurrence of the SDH with a maximum size of 15 mm, responsible for a 20 mm shift of the midline as well as a recurrence of the occipital lesion (see Figure 1D,E). An emergency mini-craniotomy centered on the previous burr-hole was performed, revealing an acute, membranous SDH that was evacuated without complication. Some membranes found in the SDH were sent for further pathological analyses (the surgical and pathological teams ensured that no tissue from the original tumoral site was mixed with these specimens to prevent any contamination). The recurrent occipital lesion was not removed during the surgery. No postoperative anticoagulant treatment was administered to the patient. Histopathological analysis of the membranes revealed the presence of tumoral cells compatible with the original occipital HGG (see Figure 2D–G). Of note, there was no cytological analysis on the patient’s CSF. The patient presented with an initial clinical improvement in the immediate postoperative period (GCS 14 due to confusion with improvement of the hemiparesis) but then presented a third clinical worsening during his hospital stay. Given the clinical worsening and the recurrence of the primary lesion as well as the presence of tumoral cells in the evacuated SDH, no further surgery was proposed, and the patient was transferred to our palliative care unit where he sadly passed away a few hours after his admission.

## 3. Discussion

In this article, we present a case of recurrent, spontaneous SDH after HGG resection. Histopathological analyses revealed that HGG cells were present in the membranes comprising the recurrent hematoma.

Spontaneous hematoma, being intra- or extra-parenchymal, can be the first clinical manifestation of brain tumors. This mode of presentation has been described for meningiomas, dural metastases and HGG [15,16,17,18,19]. Indeed, HGG has been reported to mimic non-traumatic intracranial bleeding (such as SDH), leading to preoperative misdiagnosis and intraoperative modification of treatment strategy [20,21]. While spontaneous extra-cranial bleeding has also, but less frequently, been reported after HGG resection [11,12,13], and while Wei et al. reported HGG development after chronic SDH evacuation [22], direct histological observation of HGG tumoral cells in hematomas has not been previously reported in the literature to the best of our knowledge. Indeed, in this case, we found typical HGG cells (with the same histopathological characteristics as the initial lesion) in the membranes of the recurrent SDH. This raises the question of whether the formation of the SDH occurred before or after the spread of HGG cells. While no definite conclusion can be drawn, two hypotheses seem to prevail regarding the temporality of both conditions.

On the one hand, the occurrence of a spontaneous SDH might have led to the spreading of tumoral cells outside the initial resection site and into the subdural space. In this case, the SDH would have been responsible for the spread of the tumor (allowing for cellular invasion of the surrounding tissues), comparable to CSF in the case of leptomeningeal spreading of tumoral lesions [23]. It is important to note that there was no cytological analysis of the patient’s CSF in this case, and that we cannot rule out a role for this system in the dissemination of tumoral cells. On the other hand, the tumoral spread into the subdural space might have occurred first, resulting in the development of the SDH due to an angiodysplastic reaction of the surrounding tissues or the bleeding of abnormal, tumor-derived vessels in the newly formed membranes [7,24]. Finally, we cannot rule out a combination of the two hypotheses, with the tumoral cells being responsible for, or facilitating, SDH formation and the bleeding, in turn, facilitating further spreading of tumoral cells.

Importantly, the fact that the SDH showed fast recurrence after evacuation seems at least partly to point towards a role for the tumoral cells and associated membranes in the formation of the hematoma. Indeed, in our case, spontaneous bleeding reoccurred despite surgical evacuation of the first SDH, and this reoccurrence raises the possibility that tumoral dissemination was already present at the time of the first bleeding.

This work is not devoid of limitations. The fact that the first SDH was not histologically analyzed prevents us from concluding whether tumor cells were already present in the components of this hematoma. Moreover, the generalizability of one single case report to all HGG-related hematomas is not possible and further research is therefore warranted (1) to confirm the presence of tumoral cells in HGG-related hematomas as well as the frequency of this phenomenon, and (2) to identify the correct mechanism for HGG tumoral cells’ dissemination in those hematomas. Of note, the impossibility of our patient benefitting from any adjuvant oncological therapy (either chemotherapy or radiotherapy) due to the hemorrhagic complications might have further favored the spread of tumoral cells from the initial resection site.

## 4. Conclusions

In this article, we report a very rare case of recurrent SDH after HGG resection, in which histopathological analyses of SDH-derived membranes showed clear signs of tumoral cells invasion of the subdural space. These observations raise the question of whether any HGG-associated hematoma requiring surgical evacuation should be analyzed for the presence of tumor cells. This is of utmost importance as leptomeningeal invasions result in worse prognosis [23] and might ultimately lead to a modification of HGG patients’ management (notably a change in chemotherapy or towards palliative care).

## Data Availability

The data presented in this study are available on request from the corresponding author.

## References

[1] Ostrom Q.T., Price M., Neff C., Cioffi G., Waite K.A., Kruchko C., Barnholtz-Sloan J.S. (2022). CBTRUS Statistical Report: Primary Brain and Other Central Nervous System Tumors Diagnosed in the United States in 2015–2019. Neuro-Oncology.

[2] Louis D.N., Perry A., Burger P., Ellison D.W., Reifenberger G., von Deimling A., Aldape K., Brat D., Collins V.P., Eberhart C. (2014). International Society of Neuropathology-Haarlem Consensus Guidelines for Nervous System Tumor Classification and Grading. Brain Pathol..

[3] Ostrom Q.T., Patil N., Cioffi G., Waite K., Kruchko C., Barnholtz-Sloan J.S. (2020). CBTRUS Statistical Report: Primary Brain and Other Central Nervous System Tumors Diagnosed in the United States in 2013–2017. Neuro-Oncology.

[4] Wakai S., Yamakawa K., Manaka S., Takakura K. (1982). Spontaneous Intracranial Hemorrhage Caused by Brain Tumor: Its Incidence and Clinical Significance. Neurosurgery.

[5] Broka A., Hysenaj Z., Sharma S., Rehmani R. (2021). Lion in Sheep’s Clothing: Glioblastoma Mimicking Intracranial Hemorrhage. Cureus.

[6] Chan Z.W.Y., Gallo P. (2018). Chronic Calcified Subdural Haematoma Found after Presentation of Symptomatic Glioma. BMJ Case Rep..

[7] Al-Mousa A., Al-Dwairy S., Dajani N., Sami R., Shtaya A. (2023). Giant Cell Glioblastoma Multiforme Presents as Acute Pathological Nontraumatic Subdural Haematoma. Br. J. Neurosurg..

[8] Lee J., Kim M.-S., Kim Y.Z. (2019). Extensive Pachymeningeal Dissemination of Glioblastoma Mimicking Chronic Subdural Hematoma: A Case Report. Brain Tumor Res. Treat..

[9] Adegboyega G., Kanmounye U.S., Petrinic T., Ozair A., Bandyopadhyay S., Kuri A., Zolo Y., Marks K., Ramjee S., Baticulon R.E. (2021). Global Landscape of Glioblastoma Multiforme Management in the Stupp Protocol Era: Systematic Review Protocol. Int. J. Surg. Protoc..

[10] Honeyman S.I., Owen W.J., Mier J., Marks K., Dassanyake S.N., Wood M.J., Fairhead R., Martinez-Soler P., Jasem H., Yarlagadda A. (2024). Multiple Surgical Resections for Progressive IDH Wildtype Glioblastoma—Is It Beneficial?. Acta Neurochir..

[11] Paisan G.M., Buell T.J., Raper D., Asthagiri A. (2017). Lumbosacral Subdural Hematoma After Glioblastoma Multiforme Resection: Possible Radiographic Evidence for the Downward Migration of Intracranial Blood. World Neurosurg..

[12] Koutsouras G.W., Amsellem A., Richardson T., Babu H. (2021). Multifocal Spinal Glioblastoma and Leptomeningeal Carcinomatosis in an Elderly Male with Hydrocephalus and Myelopathy. Surg. Neurol. Int..

[13] Cai J., Zhang Y., Bai X., Li S., Chen J., Chen R., Lin H., Huang S. (2014). Postoperative Hemorrhage in an Elderly Patient with a Glioblastoma Multiform and a Calcified Chronic Subdural Hematoma. World J. Surg. Oncol..

[14] Kaptein F.H.J., Stals M.A.M., Kapteijn M.Y., Cannegieter S.C., Dirven L., van Duinen S.G., van Eijk R., Huisman M.V., Klaase E.E., Taphoorn M.J.B. (2022). Incidence and Determinants of Thrombotic and Bleeding Complications in Patients with Glioblastoma. J. Thromb. Haemost..

[15] Kunii N., Morita A., Yoshikawa G., Kirino T. (2005). Subdural Hematoma Associated with Dural Metastasis-Case Report. Neurol. Med. Chir..

[16] Di Vitantonio H., De Paulis D., Alessandro R., Sara M., Juan G.R. (2014). Meningioma Associated with Acute Subdural Hematoma: A Review of the Literature. Surg. Neurol. Int..

[17] Wang A.M., Chinwuba C.E., O’Reilly G.V., Kleefield J. (1985). Subdural Hematoma in Patients with Brain Tumor: CT Evaluation. J. Comput. Assist. Tomogr..

[18] Richard S.A., Ye Y., Li H., Ma L., You C. (2018). Glioblastoma Multiforme Subterfuge as Acute Cerebral Hemorrhage: A Case Report and Literature Review. Neurol. Int..

[19] Kotwica Z., Zawirski M. (1986). Subdural Hematoma Caused by Cerebral Tumors. Zentralbl. Neurochir..

[20] Khanna R., Brahimaj B., Tchalukov K., Byrne K., Adogwa O., Jhaveri M., Byrne R. (2019). A Case of Recurrent Gliosarcoma Mimicking Subdural Hematoma. Interdiscip. Neurosurg..

[21] Mikhalkova A., Hoffermann M. (2020). Extensive Subdural Spread of a Glioblastoma Associated with Subdural Hygroma: Case Report. J. Surg. Case Rep..

[22] Wei W., Yang T., Liu X., Li L., Fan Y. (2025). Gliomagenesis Following Chronic Subdural Hematoma: A Case Report. Exp. Ther. Med..

[23] Machado M., Salcman M., Kaplan R.S., Montgomery E. (1985). Expanded Role of the Cerebrospinal Fluid Reservoir in Neurooncology: Indications, Causes of Revision, and Complications. Neurosurgery.

[24] Braun E.M., Burger L.J., Schlang H.A. (1973). Subdural Hematoma from Metastatic Malignant Disease. Cancer.

